# Proof of concept non-invasive estimation of peripheral venous oxygen saturation

**DOI:** 10.1186/s12938-017-0351-x

**Published:** 2017-05-19

**Authors:** Musabbir Khan, Chris G. Pretty, Alexander C. Amies, Joel Balmer, Houda E. Banna, Geoffrey M. Shaw, J. Geoffrey Chase

**Affiliations:** 1 Centre for Bioengineering, Department of Mechanical Engineering, University of Canterbury, Private Bag 4800, Christchurch, 8140 New Zealand; 2 Department of Intensive Care Unit, Christchurch Hospital, Private Bag 4710, Christchurch, 8140 New Zealand

**Keywords:** Non-invasive, Pulse oximeter, Photoplethysmograph, SvO_2_ estimation, Artificial modulation

## Abstract

**Background:**

Pulse oximeters continuously monitor arterial oxygen saturation. Continuous monitoring of venous oxygen saturation (SvO_2_) would enable real-time assessment of tissue oxygen extraction (O_2_E) and perfusion changes leading to improved diagnosis of clinical conditions, such as sepsis.

**Methods:**

This study presents the proof of concept of a novel pulse oximeter method that utilises the compliance difference between arteries and veins to induce artificial respiration-like modulations to the peripheral vasculature. These modulations make the venous blood pulsatile, which are then detected by a pulse oximeter sensor. The resulting photoplethysmograph (PPG) signals from the pulse oximeter are processed and analysed to develop a calibration model to estimate regional venous oxygen saturation (SpvO_2_), in parallel to arterial oxygen saturation estimation (SpaO_2_). A clinical study with healthy adult volunteers (*n* = 8) was conducted to assess peripheral SvO_2_ using this pulse oximeter method. A range of physiologically realistic SvO_2_ values were induced using arm lift and vascular occlusion tests. Gold standard, arterial and venous blood gas measurements were used as reference measurements. Modulation ratios related to arterial and venous systems were determined using a frequency domain analysis of the PPG signals.

**Results:**

A strong, linear correlation (*r* ^*2*^ = 0.95) was found between estimated venous modulation ratio (R_Ven_) and measured SvO_2_, providing a calibration curve relating measured R_Ven_ to venous oxygen saturation. There is a significant difference in gradient between the SpvO_2_ estimation model (SpvO_2_ = 111 − 40.6*R) and the empirical SpaO_2_ estimation model (SpaO_2_ = 110 − 25*R), which yields the expected arterial-venous differences. Median venous and arterial oxygen saturation accuracies of paired measurements between pulse oximeter estimated and gold standard measurements were 0.29 and 0.65%, respectively, showing good accuracy of the pulse oximeter system.

**Conclusions:**

The main outcome of this study is the proof of concept validation of a novel pulse oximeter sensor and calibration model to assess peripheral SvO_2_, and thus O_2_E, using the method used in this study. Further validation, improvement, and application of this model can aid in clinical diagnosis of microcirculation failures due to alterations in oxygen extraction.

## Background

Arterial oxygen saturation (SaO_2_) and venous oxygen saturation (SvO_2_) are the two basic parameters used to assess the oxygen delivery process and monitor oxygen extraction (O_2_E). These two parameters are important for the analysis of whole body oxygen circulation. O_2_E data could provide an indicator of the adequacy of local tissue perfusion, to aid in the early diagnosis of microcirculatory dysfunction in medical conditions, such as in sepsis and cardiogenic shock. Thus, continuous monitoring of both SaO_2_ and SvO_2_ would be useful for haemodynamic and perfusion management in clinical settings, according to recent clinical consensus statements [1].

SaO_2_ can be non-invasively and reliably estimated by pulse oximeters (SpaO_2_), using photoplethysmograph (PPG) signals produced by an optical sensor, typically mounted on a finger, toe, or ear-lobe, to detect blood volume changes. The PPG waveform results from the pulsatile variation in the tissue optical density produced by the pulsation of arterial blood [2, 3]. Conventional pulse oximetry relies on the pulsatile nature of arterial blood and differential absorption of oxyhaemoglobin and de-oxyhaemoglobin at red (RD) and infrared (IR) wavelengths to estimate SpaO_2_ [4, 5].

Venous blood in the periphery is typically non-pulsatile in nature. Being dependent on pulsatile blood volume changes to make measurements, conventional pulse oximeter sensors can only determine SpaO_2_. Thus, SvO_2_ estimation cannot be provided by conventional pulse oximeters (SpvO_2_). Currently, no available commercial equipment provides continuous, non-invasive measurement or estimate of SvO_2_.

Reliable SvO_2_ monitoring is an important element of perfusion monitoring and a necessary measure to determine tissue oxygen extraction capability, which can be used as a clinical marker of microcirculatory failures. In addition, continuous monitoring of the difference between SaO_2_ and SvO_2_ would enable the tracking of alterations in tissue perfusion, in real-time. These alterations are very common in sepsis patients [6]. Thus, continuous and simultaneous SpaO_2_ and SpvO_2_ estimation by pulse oximeter could be used as an indicator of tissue O_2_E and perfusion changes in response to patient condition or treatment.

Prior studies have investigated non-invasive methods using PPG and near infrared spectroscopy (NIRS) sensors to assess SpvO_2_. However, the results of these studies have not been conclusive, or have not been validated against a reliable measurement. Two previous studies attempted to assess SpvO_2_ by inducing pulsations to the venous system, but failed to provide physiologically realistic values of SpvO_2_ [7, 8]. Some studies reported physiologically realistic SvO_2_ estimates, but did not validate their results against gold standard measurements [9–12]. Other studies used reference measurement for comparison, but cannot provide continuous and simultaneous measurement of both SpaO_2_ and SpvO_2_ [13, 14]. These limitations of prior studies reduce the clinical applicability of their methods.

**Fig. 1:**
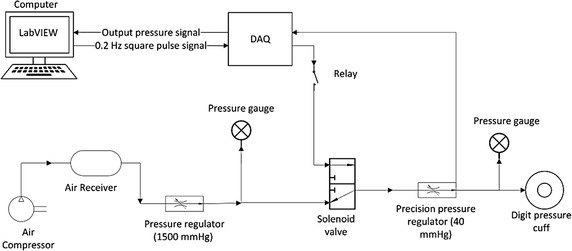
APG system functional block diagram illustrating overall operation

Venous walls are significantly thinner and less elastic than arterial walls. In particular, the veins are up to 10–20 times more compliant compared to arteries under low pressure [15–17]. With relatively small changes in pressure, the circulating blood inside the much more compliant veins experiences large volume changes compared to the arteries [16, 18]. This large compliance difference is what causes the venous blood of the oesophagus regions to modulate at respiratory frequency seen in previous studies [16, 18], and is illustrated in Fig. 1. Hence, this compliance difference can be exploited to artificially induce modulations at respiratory frequency and low pressure in the venous system, without disturbing arterial blood flow [19], which can be measured using PPG.

This study presents the initial proof of concept for a novel pulse oximeter method to assess peripheral SpvO_2_, in addition to SpaO_2_, leveraging this arterial-venous compliance difference. An artificial pulse generation (APG) system was developed to artificially modulate the peripheral venous blood using a digit pressure cuff. These modulations are detected by a pulse oximeter sensor and the resultant PPG signals are extracted for analysis. The measured transmission from the PPG signal related to venous blood and the SvO_2_ from gold standard blood gas measurement were used to develop a calibration curve. The main outcome of this study is a proof of the concept and a calibration curve to estimate SpvO_2_ and accuracy of the sensor.

## Methods

### APG system development and operation

**Fig. 2:**
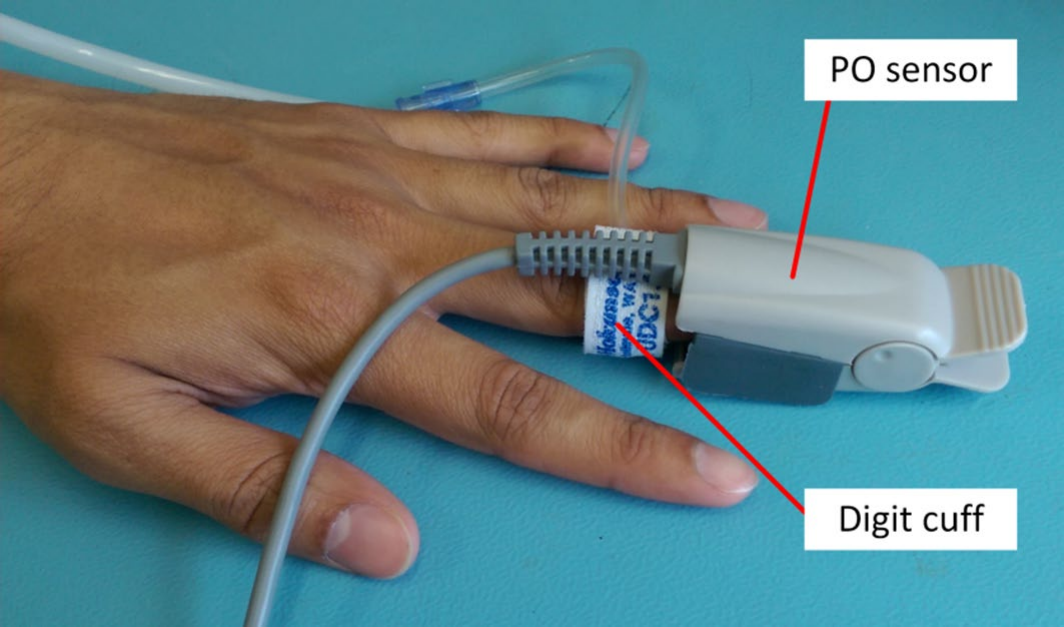
Digit cuff placement adjacent to the PPG sensor

An APG system was developed in this study to exploit the significant arterial-venous compliance difference to induce artificial pulsations predominantly in the venous compartment. The complete APG system is shown schematically in Fig. 1. This system uses a pneumatic UDC2.5 (D.E. Hokanson Inc., Bellevue, WA, USA) digit cuff placed at the intermediate phalanges of the middle finger, as shown in Fig. 2, to mechanically modulate venous blood in the finger. By periodically inflating and deflating the digit cuff, an artificial respiration-like pulse can be induced onto the venous blood of the finger.

Inflation/deflation of the digit cuff is controlled by a 3-way solenoid valve (EVT 307-5DZ-02F-Q, SMC, Noblesville, IN, USA). Operation of the solenoid valve is managed by a PC running LabVIEW through a NI cDAQ-9172 (National Instruments, Austin, TX, USA) multifunction data acquisition device (DAQ). The solenoid is switched on and off with square pulses at 0.2 Hz and 50% duty cycle. The 0.2 Hz frequency is close to typical respiratory frequencies to create artificial venous blood modulations, like those previously reported by Walton et al. [9] when detecting natural venous blood modulations in the oesophagus using a PPG sensor.

A precision pressure regulator (AS1002F, SMC, Noblesville, IN, USA) was used to control the pressure of air supplied to inflate the cuff at 40–50 mmHg, which is approximately 50% below typical diastolic arterial pressures. Thus, this cuff compression pressure will predominantly effect the venous compartment. For the deflation portion of the pulse, the air in the cuff was exhausted to the atmosphere with the solenoid valve turning off.

### Study cohort

Ethics approval for this study was granted by the Human Disability an Ethics Committee, New Zealand (Reference: 15/CEN/141). This study was conducted in the intensive care unit (ICU) of St George’s Hospital, Christchurch. Eight healthy adult, male, volunteers (aged 23–37 years) with no pre-existing medical conditions were recruited, and provided signed, informed consent. Table 1 provides subject demographics including age, sex, height, weight, body mass index (BMI), resting blood pressure (BP), and resting heart rate (HR). This study was an initial proof of concept testing using healthy adult volunteers and was not about testing full range of people, age, gender or other condition.

**Table 1:** Subject demographics for this study

Subject	Age (years)	Sex	Height (m)	Weight (kg)	BMI	Resting BP systolic/diastolic (mmHg)	Resting HR (bpm)
1	30	M	1.75	74.40	24.16	125/68	52
2	24	M	1.78	69.00	21.78	106/49	73
3	37	M	1.83	75.00	22.40	120/63	66
4	23	M	1.71	70.00	23.94	131/78	76
5	24	M	1.68	55.00	19.49	117/56	65
6	27	M	1.68	71.50	25.51	120/70	68
7	30	M	1.80	93.00	28.70	134/87	69
8	25	M	1.70	65.00	22.49	109/55	68

### Equipment set up

**Fig. 3:**
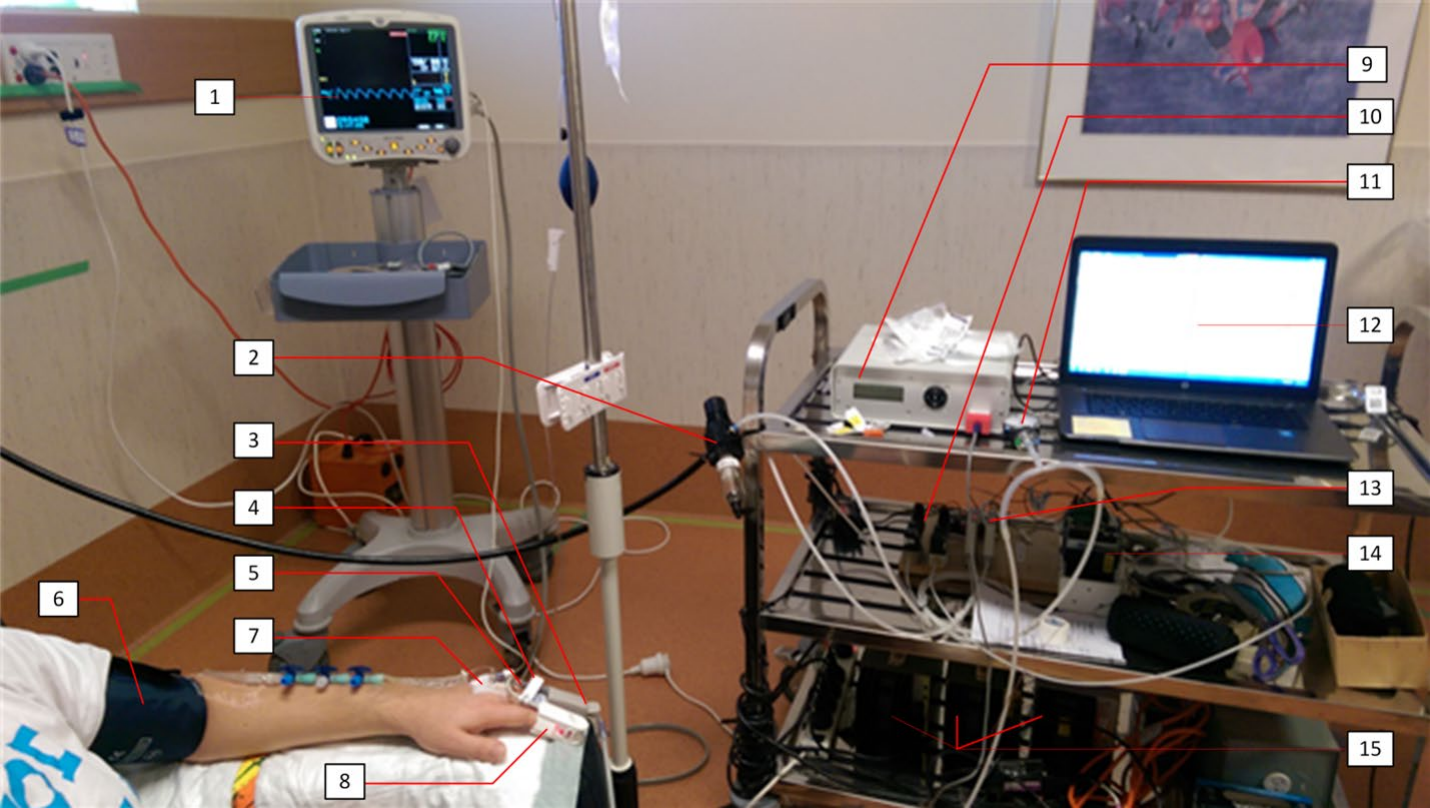
Typical equipment set up for this study. Key components are labelled. *1* dash monitoring screen, *2* air supply and regulator, *3* PO sensor, *4* thermocouple sensor, *5* digit pressure cuff, *6* blood pressure cuff, *7* venous line and catheter, *8* masimo SET sensor, *9* PO system, *10* solenoid vales, *11* pressure gauge, *12* PC running LABVIEW, *13* ITV001 pressure regulator, *14* NI DAQ system, *15* power supplies

The equipment for this experiment is shown in Fig. 3 and was set up as follows:

- An arterial catheter (20 or 22 g) was inserted into the left radial artery under sterile conditions using a small amount (0.2 ml) of subcutaneous local anaesthetic (lidocaine 1%). Similarly, a 20–22 g IV catheter was inserted into a vein on the back of the left hand. Both catheters were connected to an ‘intraflow’ device, which delivered about 3 ml per hour of 0.9% saline through the arterial catheter. A portable i-Stat1 blood gas analyser (Abbott, Princeton, NJ, USA) was employed to analyse catheter drawn blood samples.
- A standard transmission mode sensor (model: 320701001, Biometric Cables, Guindy, Chennai, India), connected to the custom pulse oximeter (PO) system, was clipped to the index/middle finger of the left hand.
- A Masimo (Masimo Corporation, Irvine, CA, USA) SET pulse oximeter sensor was clipped to the ring finger of the left hand and connected to a portable DASH 5000 (GE Healthcare, Chicago, Illnois, USA) monitoring screen as a reference measurement for SpaO_2_ and HR.
- A thermocouple was attached next to the PO sensor on the skin surface to continuously monitor digit skin temperature.
- A conventional blood pressure cuff was wrapped around the participant’s left forearm to make blood pressure measurements and perform vascular occlusion test (VOT), according to the experimental protocol.

### Experimental protocol

Subjects were asked to refrain from smoking and strenuous physical activities for at least 4 h prior to the experiment. During the study, subjects were comfortably seated, while resting their left arm on a flat surface at approximately the same height as their heart and with minimum movement. During the study, 3 tests were performed to achieve a wide range of SvO_2_ values and provide a valid dataset.

- At the beginning of the study, resting HR, resting BP, height, weight, and BMI were recorded, as presented in Table 1. Two minutes of baseline PPG data were recorded without any APG activation.
- Subjects were asked to perform the following tests as part of the study protocol:
  - Test 1: Hold left arm at chest level, referred to as baseline.
  - Test 2: Raise left arm above the head for 10 min.
  - Test 3: Allow pressure cuff induced vascular occlusions for 4–6 min using pressures of 170 mmHg, which are above typical systolic pressures to stop all flow to the hand.
- 2 ml sample of blood was drawn during each test as follows:
  - Test 1: 1× arterial sample, 1× venous sample after 1 min of APG activation.
  - Test 2: 1× arterial sample (Subjects 1–5 only) and 1× venous sample at 4 min after the arm was raised.
  - Test 3: 1× venous sample immediately before the pressure cuff was released, and 1× venous sample at 30 s into post occlusion.

Up to a total of 8 blood samples were drawn from each participant. Each blood sample was approximately 2 ml and maximal total blood volume approximately 16 ml. The catheters were flushed with 0.9% saline (1–3 ml) after each blood draw. Subjects 7 and 8 had 2× venous samples taken during Test 1 to get more baseline measurements and increase the number of overall data samples for analysis.

**Fig. 4:**
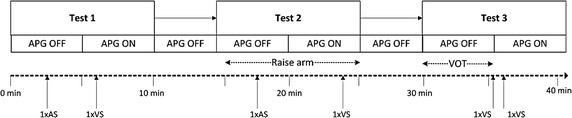
Timeline for the overall experimental protocol, including APG operations and blood sampling times for each test. Participants were not asked to complete more than 2 runs through the protocol. *AS* refers to arterial blood sample. *VS* refers to venous blood sample

- APG operation and blood sampling times for the tests are illustrated in Fig. 4:

For Subjects 1–5, SaO_2_ measurements did not vary more than ±1.02% of the baseline measurements (Test 1) for any tests. Since SaO_2_ unlikely to vary with any change in local conditions for a healthy adult, only one arterial sample was drawn during Test 1 as a reference sample for Subjects 6–8. Thus, no other arterial samples were obtained during Tests 2 and 3 for Subjects 6–8 to simplify the study and reduce burden, since the primary goal is to assess the SvO_2_ provided versus gold standard measures of the same SvO_2_.

Test 2 was investigated for Subjects 1–5 only to detect any change in SvO_2_ using the PO sensor and APG system. When the arm is raised above heart level, the mean arterial blood pressure and flow is reduced, and thus a drop in SvO_2_ can occur. During testing, it proved difficult to reliably draw blood samples with the lowered venous flow and pressure, meaning a range of induced oxygenation levels could not be effectively achieved. Thus, Test 3 was developed to improve reliability and ease of blood sampling for a similar induced change, where it is important to note that inducing a range of oxygenation levels is the only necessity in this initial proof of concept study whose goal is to provide initial validation of the potential to accurately capture a wide range of venous oxygenation levels.

More specifically, Test 3 was conducted as an alternate technique to cause a more repeatable and more easily induced significant drop in SvO_2_. Several minutes of ischemia is likely to induce a large reduction in overall oxygen saturation, including SvO_2_, in the ischemic region. It should be noted that Test 3 was repeated for Subjects 6–8 for 4 and 6 min of vascular occlusions, while Subject 5 being the first for Test 3 was only tested for 4 min of occlusion as the protocol was still being developed. Thus, Test 3 not only induced a larger SvO_2_ change but was also more reliable, repeatable, and easier to implement compared to Test 2.

### PPG acquisition and sampling

PPG finger sensor control and data acquisition were managed with the PO system, which includes serial communication and a graphical interface. The PO system is based on the CY8CKIT-050 PSoC 5LP Development Kit (Cypress Semiconductor, San Jose, CA, USA) that has an ARM Cortex M3 microcontroller. The PPG sensor uses 660 and 940 nm wavelengths for the RD and IR LEDs, respectively, and a very sensitive photodetector (PD). Feedback control was used by the PO system microcontroller to adjust LED intensities specific to finger thickness.

Light energy received at the PD was converted to current signals and passed through a two stage transimpedance amplifier for signal conditioning. A de-multiplexer splits the conditioned PPG signals so that the RD and IR PPGs are processed independently. Analog PPG signals in the range 0–5 V were sampled at 50 Hz by a 16-bit analog-to-digital converter (ADC) on the development board. Sampled data were sent to a PC via serial communication and recorded for offline signal processing in MATLAB (R2014a, MathWorks, Natick, MA, USA).

### Data processing and analysis

A two-stage digital filter system was implemented to extract the PPG signals of interest from the raw and amplified PPG data. Zero phase (forward–reverse) filtering was applied at each filter stage to reduce phase distortion and maintain PPG waveform shape.

The first filter stage was a 170th order, equiripple, low-pass, FIR filter with a cut-off frequency of 10 Hz and attenuation of 50 dB to effectively remove any unwanted, high frequency noise from the PPG signals [20]. The second filter stage had three parallel filters to separately extract high frequency (AC), APG modulated frequency (APG), and very low frequency (DC) signals. An empirically derived 4th order band-pass, Butterworth, IIR filter extracted AC signals, such as the cardiac frequencies, with pass band frequencies of 0.67–4.5 Hz. An empirically derived 12th order band-pass, Butterworth, IIR filter extracted APG signals with pass band frequencies of 0.15–0.67 Hz. An empirically derived 6th order low-pass, Butterworth, IIR filter with a cut-off frequency of 0.15 Hz was used to extract DC signals between 0 and 0.15 Hz.

A Fast Fourier Transform (FFT) analysis was used to determine the relative power in each part of the PPG signal to calculate the “modulation ratios” (R values):

1 $$R_{Art} = \frac{{\left(|AC|_{HR} /|DC|_{0 Hz} \right)_{RD} }}{{\left(|AC|_{HR} /|DC|_{O Hz} \right)_{IR} }}$$

2 $$R_{Ven} = \frac{{\left(\left| {APG} \right|_{0.2 Hz} /|DC|_{0 Hz} \right)_{RD} }}{{\left(\left| {APG} \right|_{0.2 Hz} /|DC|_{O Hz} \right)_{IR} }}$$

where,

- R_Art_ is the modulation ratio related to arterial blood.
- R_Ven_ is the modulation ratio related to venous blood.
- |AC|_HR_ is the peak Fourier magnitude of the AC signal, related to cardiac frequency.
- |APG|_0.2 Hz_ is the peak Fourier magnitude at 0.2 Hz of the APG signal.
- |DC|_0 Hz_ is the peak Fourier magnitude at 0 Hz frequency of the DC signal.

**Fig. 5:**
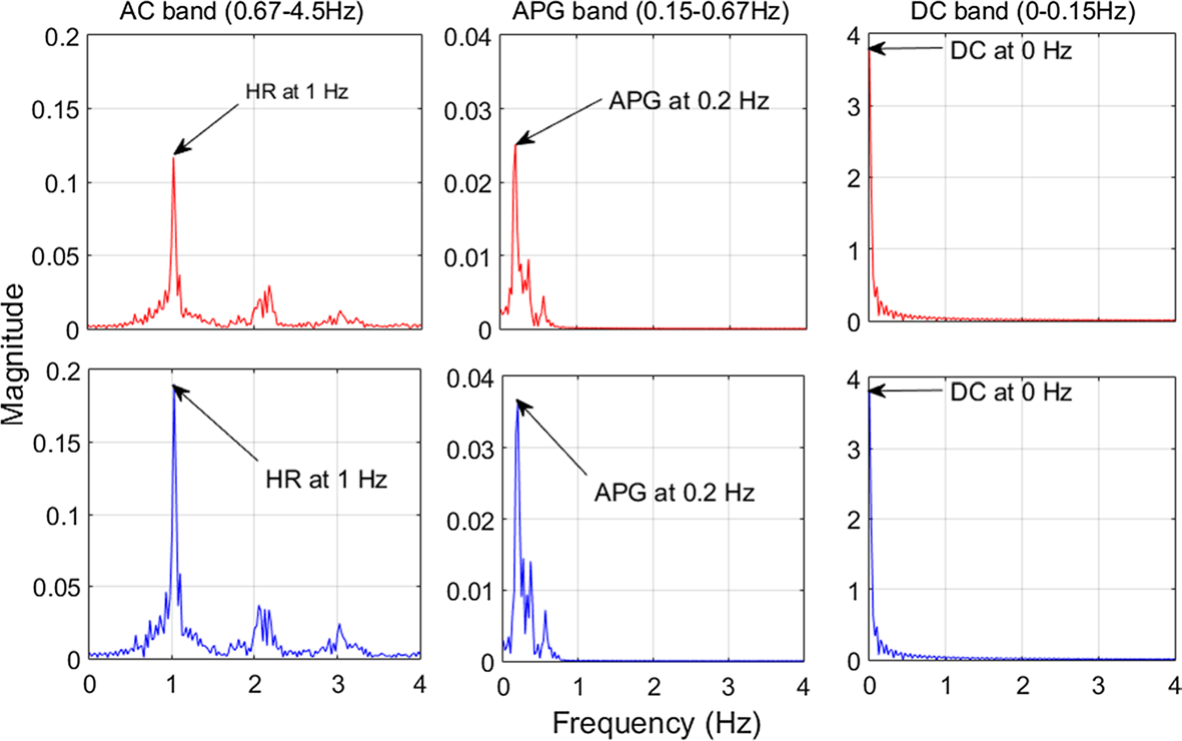
Frequency power spectrums of the AC, APG, and DC filter bands for Subject 7 from Test 1. *Top panels*: RD and *bottom panels*: IR

The fundamental frequency associated with the peak magnitude was identified from the frequency power spectra of AC, APG, and DC offset signals, as shown in Fig. 5. Equations 1 and 2 were then used to compute values of R_Art_ and R_Ven_. Frequency power spectra were obtained using the FFT on PPG data blocks of 20 s duration (n = 1000 samples), yielding a frequency resolution of 0.025 Hz. Each 20 s block corresponded to a blood sample, enabling comparison between measured and estimated saturation values. Blocks of 20 s were short enough to allow analysis of transient effects in Test 3, while still providing good frequency resolution.

A linear model was fitted to the calculated R_Ven_ values and measured blood gas SvO_2_, and the coefficient of determination (*r* ^2^) was computed to assess the strength of their correlation. The linear model was then used as the calibration curve to estimate SpvO_2_ for each R_Ven_. The Wilcoxon signed rank test was used to compare paired values of measurements, including R_Ven_ and R_Art_ for each subject, over the experimental cohort. *p* values of *p* < 0.05 were considered statistically significant.

Error propagation analysis, using linear and non-linear combinations [21], was implemented to calculate uncertainties for measured SvO_2_ $\left( {\delta {\text{SvO}}_{2} } \right)$ and R_Ven_ $\left( {\delta {\text{R}}_{\text{Ven}} } \right)$. The clinical data defined in the Abbott i-Stat1 device manual [22] and the uncertainty of the PO sensor for SpaO_2_ measurement [23, 24] were used in the analysis.

## Results

### Detection of AC and APG signals from PPG

**Fig. 6:**
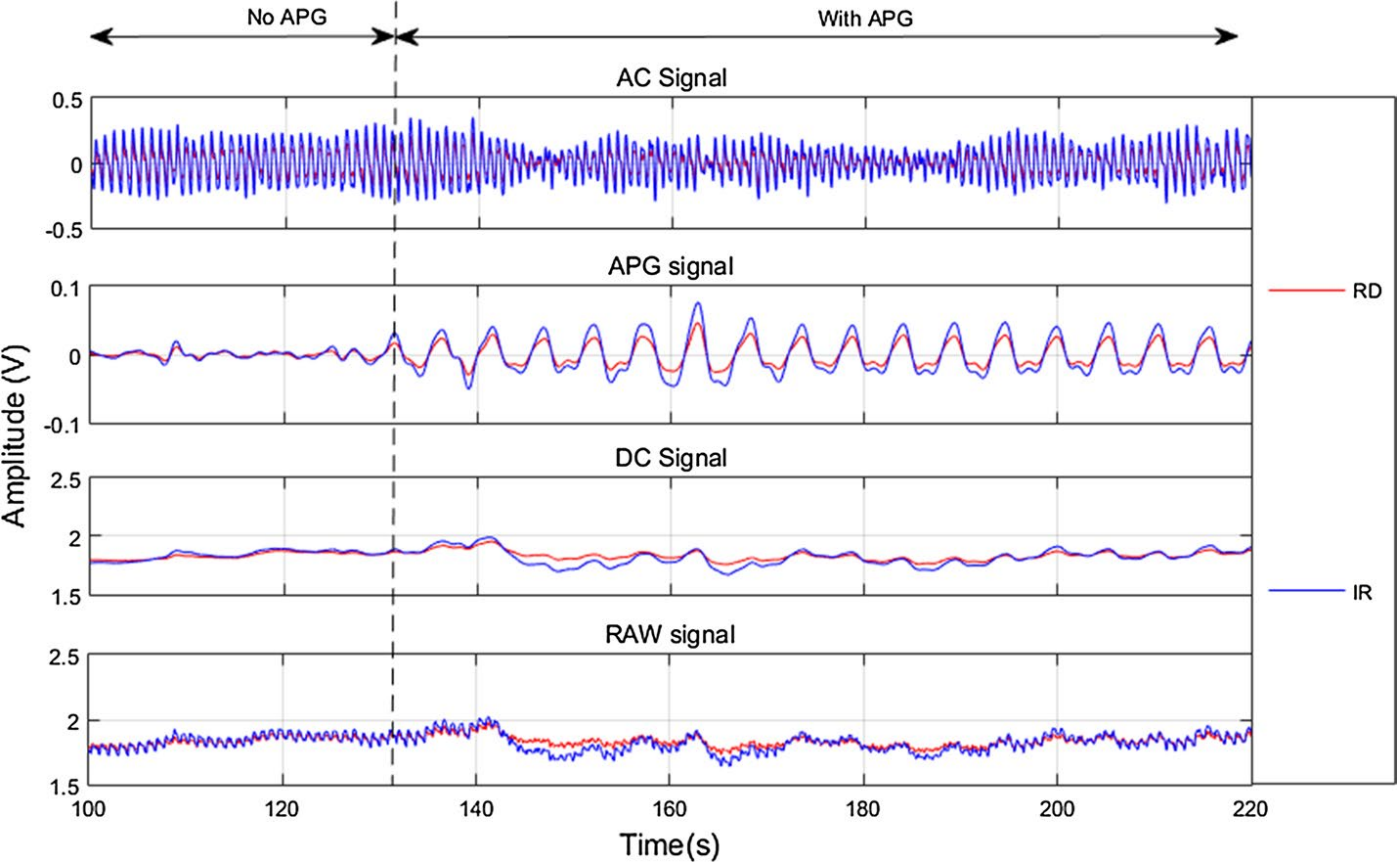
Baseline PPG signals showing pre APG and post APG phases for Subject 7; top-bottom AC signal, APG signal, DC Signal, and RAW signal

Figure 6 shows an example of the post-processed PPG signals detected by the PO sensor, in the time domain, during Test 1. During the first 120 s of Test 1, the APG signal was effectively constant since the APG system was not activated, while the AC signal pulsed due to the cardiac cycle. Upon activation of the APG system at approximately 130 s, the APG signal showed a period oscillation with an amplitude of up to 0.06 V.

### Correlation between R_Ven_ and SvO_2_

**Fig. 7:**
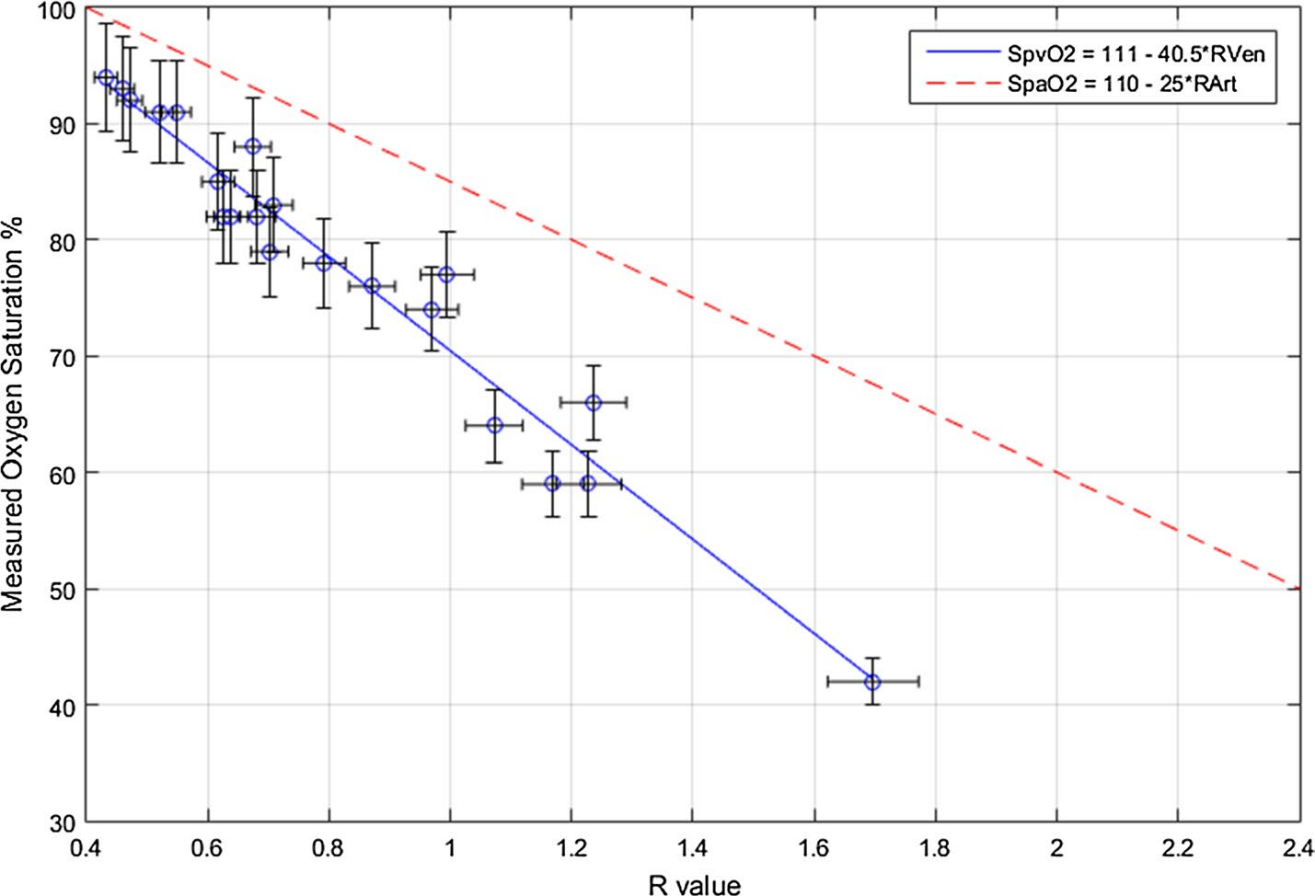
Correlation (*r* ^*2*^ = 0.95) between all estimated R_Ven_ and measured SvO_2_ samples across the whole cohort from the 3 tests. The *solid blue line* is the proposed SpvO_2_ calibration model and the *dashed red line* is the empirical SpaO_2_ calibration model related to R_Art_ [25]

Table 2 presents all the estimated R_Ven_ values and measured blood gas SvO_2_ samples, including their estimated uncertainties, for each subject in all the tests (*n* = 21). The correlation between these two measurements is shown in Fig. 7. A strong, negative correlation is found between the two measurements, with *r* ^*2*^ = 0.95. The linear model fitted through the data points shows a good association between R_Ven_ and SvO_2_ and explains all but 5% of the variation. In addition, the gradient (−40.5%) of this model is different to the gradient (−25.0%) of the empirical SpaO_2_ estimation model commonly used in pulse oximeter estimation [25] and shown on Fig. 7 for comparison.

**Table 2:** Estimated R_Ven_, measured SvO_2_, and related uncertainties for each subject from the whole study

Subject	R_Ven_	$\delta {\text{R}}_{\text{Ven}}$	SvO_2_%	$\delta {\text{SvO}}_{2}$
1	0.64	0.03	82.00	3.99
	0.70	0.03	79.00	3.85
2	0.55	0.02	91.00	4.43
3	0.63	0.03	82.00	3.99
	0.52	0.02	91.00	4.43
4	0.87	0.04	76.00	3.70
	0.97	0.04	74.00	3.60
5	0.43	0.02	94.00	4.58
	0.46	0.02	93.00	4.53
	1.07	0.05	64.00	3.12
6	0.68	0.03	82.00	3.99
	1.23	0.05	59.00	2.87
	1.70	0.07	42.00	2.05
7	0.71	0.03	83.00	4.04
	0.62	0.03	85.00	4.14
	0.79	0.03	78.00	3.80
	1.24	0.05	66.00	3.21
8	0.67	0.03	88.00	4.29
	0.47	0.02	92.00	4.48
	0.99	0.04	77.00	3.75
	1.17	0.05	59.00	2.87

Most of the data points are in close proximity to the fitted linear model. The data point at R = 1.7, SvO_2_ = 42% could have some influence on the liner fit due to its distance from the other points. In this case, exclusion of this point still shows a strong correlation (*r* ^2^ = 0.92) between the two measured and calculated values, indicating the point does not significantly affect the overall correlation.

### Measured and estimated data

Table 3 summarizes all measured and estimated oxygen saturation values, including their paired differences, and R values for the three tests. Using the empirical pulse oximeter calibration equation for SpaO_2_, shown in Fig. 7, the median difference between SaO_2_ and SpaO_2_ was 0.65 [Inter Quartile Range (IQR) −0.98 to 1.51%] . The median difference between SvO_2_ and SpvO_2_, determined using the calibration equation derived for SvO_2_ developed in this study, was just 0.29 [IQR −2.05 to 1.27%] .

**Table 3:** Measured and estimated data for all the subjects across the three tests

Test	Subject	R_Ven_	SpvO_2_%	SvO_2_%	SvO_2 −_ SpvO_2_%	R_Art_	SpaO_2_%	SaO_2_%	SaO_2 −_ SpaO_2_%
1	1	0.64	84.97	82	−2.97	0.56	96.02	97	0.98
	2	0.55	88.6	91	2.4	0.52	96.91	98	1.09
	3	0.63	85.45	82	−3.45	0.58	95.62	98	2.38
	4	0.87	75.5	76	0.5	0.55	96.16	96	−0.16
	5	0.43	93.32	94	0.68	0.48	98	97	−1
	6	0.68	83.2	82	−1.2	0.47	98.16	97	−1.16
	7	0.71	82.08	83	0.92	0.63	94.34	97	2.66
		0.62	85.83	85	−0.83	0.58	–	–	–
	8	0.67	83.5	88	4.5	0.57	95.74	97	1.26
		0.47	91.71	92	0.29	0.53	–	–	–
2	1	0.7	82.35	79	−3.35	0.53	96.87	99	2.13
	3	0.52	89.73	91	1.27	0.47	98.2	97	−1.2
	4	0.97	71.53	74	2.47	0.52	97.02	96	−1.02
	5	0.46	92.2	93	0.8	0.48	97.91	97	−0.91
3	5	1.07	67.39	64	−3.39	0.69	–	–	–
	6	1.23	61.05	59	−2.05	0.7	–	–	–
		1.7	42.08	42	−0.08	0.68	–	–	–
	7	0.79	78.73	78	−0.73	0.52	–	–	–
		1.24	60.75	66	5.25	0.76	–	–	–
	8	0.99	70.5	77	6.5	0.55	–	–	–
		1.17	63.46	59	−4.46	0.62	–	–	–
Median		0.7	82.35	82	0.29	0.55	96.77	97	0.65
Interquartile range		0.62 to 0.99	70.50 to 85.83	74.00 to 88.00	−2.05 to 1.27	0.52 to 0.62	95.81 to 97.69	97.00 to 97.00	−0.98 to 1.51

Some subjects do not have measured values as explained in “Experimental protocal”. SpaO_2_ estimated using 110 − 25*R_Art_ and SvO_2_ estimated using 111 − 40.5*R_Ven_

The R_Ven_ determined across all the subjects for each protocol were lower and significantly different (*p* < 0.001) to the corresponding R_Art_, as expected. The overall median difference between estimated SpaO_2_ and SpvO_2_ across the 3 tests was 13.8%, and positive, as expected. In addition, the difference between pairs of measurements in each subject was statistically significant (*p* < 0.001).

## Discussion

This study demonstrates that peripheral SvO_2_ can be reliably and accurately estimated using the PO and APG systems, described, and the calibration curve presented. Very good correlation is found between estimated R_Ven_ and measured SvO_2_ across all the subjects in the 3 tests (*r* ^*2*^ = 0.95) across a wide range of SvO_2_ values, as shown in Fig. 7. The negative correlation is expected and in accordance to the empirically derived R_Art_ value and measured SaO_2_ in other studies [25–27]. This relationship allows determination of SvO_2_ from R_Ven_, as obtained using the APG system. Thus, the linear fit and very high *r* ^*2*^ value indicates the applicability of the metric in assessment of SvO_2_ and O_2_E in future studies.

Using this calibration model, the median (IQR) difference between pairs of measured and estimated venous oxygen saturation values was calculated to be 0.29% [−2.05 to 1.27%], which is less than the measurement error from the reference i-Stat blood gas analyzer. Using the empirical pulse oximeter calibration model, the median [IQR] difference between pairs of measured and estimated arterial oxygen saturation values was calculated to be 0.65% [−0.98 to 1.51%]. Thus, venous oxygen saturation accuracies from this pulse oximeter system are expected to be similar to those commonly obtained for arterial oxygen saturations.

The relationship between the PPG signal modulation ratio (R) and the oxygen saturation is associated with the bulk tissue’s optical properties. Modulation induced artefacts can have a measurable impact on the relative optical properties of the tissue medium being investigated using PPG [28]. Such artefacts in a limb may simulate the effects of tissue hemoglobin concentration variations of the tissues medium. These variations can result in different R values derived from PPG measurements when compared to non-modulated PPG measurements, as seen in previous studies [28, 29].

The good relationship between R_Ven_ and SvO_2_ obtained in this study suggest the low pressure external modulations caused by the APG system impacted almost exclusively the venous system, due to the significant arterial-venous compliance difference. Therefore, R_Ven_ values derived from the APG and DC offset signals are different to R_Art_ values derived from the AC and DC offset signals.

When the arm is completely occluded above systolic blood pressures, arterial blood supply to the arm is stopped. During Test 3, the arm was occluded and blood flow to the arm was restricted for several minutes. Without any perfusion it is expected that the occluded blood will slowly desaturate of oxygen over time [30–33]. The oxygen present in the occluded blood will gradually diffuse to nearby tissues due to the hypoxic condition created by low oxygen tension or PO_2_ in the occluded region. Hence, the lower SpvO_2_ and SpaO_2_ values in Test 3 and Table 3.

In Test 3, a venous sample was taken immediately before the arm pressure cuff was completely deflated to get a measure of the true ischemic, oxygen desaturated blood. The APG was activated just before releasing the pressure cuff, and thus, the first few APG pulses capture the true ischemic blood. The APG pulse amplitude of the RD signal was expected to be greater than the IR signal during such low saturation phase [34, 35]. This effect in the time domain PPG signals also has an equal effect to the PPG signals in the frequency domain. Thus, higher than baseline R_Ven_ values are observed in the Recovery 1 phase of Test 3. In addition, due to the greater inaccuracy of the wavelengths used at saturation <70% [29], the error in measurement is also larger for those R_Ven_ values.

During the occlusion phase of Test 3, the local tissue was being deprived of oxygen by the lack of perfusion. Previous studies have shown that tissues will temporarily increase extraction of oxygen from blood to return to normal tissue saturation level after ischemia [31, 36, 37]. Thus, when the cuff is released after this brief ischemia the local tissues and muscles in the arm increased extraction of oxygen, which is evident across all the subjects in Test 3, as seen in Table 3.

The recovery of oxygen desaturated blood to baseline oxygen saturation level after ischemia is very rapid during reactive hyperemia in healthy adults [30, 33]. For Subjects 5 and 6, a venous sample was taken approximately 30 s after blood flow was restored to the arm. Blood gas analysis of those samples revealed SvO_2_ had already returned to baseline level. Thus, the rapid change from low saturation to normal occurred in less than 30 s. However, this tissue oxygen restoration time can be significantly greater due to attenuated reactive hyperaemia in case of patients with sepsis as reported by previous studies [30, 38–41]. Hence, it is important to capture and track such changes in real-time for haemodynamic and perfusion monitoring.

No significant changes in SvO_2_ from baseline were observed due to lifting the arm in Test 2. These findings suggest variations in peripheral SvO_2_ and O_2_E are not influenced by reduction in peripheral arterial blood flow induced in this test. In particular, one subject in Test 2 showed elevated SvO_2_, for causes yet to be determined. However, possible reasons include; (1) increased temperature of the hand to arterialise the venous blood; (2) increased vasodilation of peripheral arteries to improve blood flow conditions; or (3) over reaction to loss of blood flow.

In addition, Test 2 posed several challenges. First, it was difficult to draw blood when the hand was held up, due to reduced venous flow and pressure. Second, a tourniquet was employed to increase venous blood volume for sampling, which may have obstructed venous flow in the periphery. Hence, Test 3 was later developed to replace Test 2 and ensure a robust, reliable, and large SvO_2_ change. However, the data from Test 2 still provided useful for development of the SpvO_2_ calibration model, and was thus kept.

### Limitations of this study

The primary limitation of this study is the relatively small number (n = 21) of data points from a male cohort used to develop the SpvO_2_ calibration curve. More data points from a gender balanced, larger, and diverse cohort will add robustness to the overall result. However, the very strong correlation obtained indicates the concept should generalize well.

In particular, this study is only a proof of concept of this pulse oximeter method to show the potential over a range of:

- SvO_2_ conditions induced to test accuracy across the physiological range.
- Peripheral blood flow levels that might be seen clinically.

The study was not about testing full range of people or sex or other conditions. This initial test was to show how the concept worked, and quantify how well. Results from this initial proof of concept study justify a complete validation trial with more subjects and a balanced cohort, in terms of age and sex.

## Conclusions

This study presents a non-invasive method to induce venous blood modulations using a pneumatic digit cuff (0.2 Hz, 40–50 mmHg inflation pressure), which can be captured by PPG. A novel calibration model was derived from venous blood modulated PPG signals and measured SvO_2_ to assess SpvO_2_. A strong, linear correlation (*r* ^*2*^ = 0.95) was found between estimated R_Ven_ and measured SvO_2_, with the median difference of 0.29% between pairs of measurement, providing a robust calibration and accurate estimate. In addition, the gradient of this model (−40.5%) is different to the gradient of the conventional pulse oximeter calibration model (−25%). The proposed SpvO_2_ estimation model can be used as a potential metric and indicator for low O_2_E, consumption, and tissue hypoxia.

Real-time estimation of peripheral SvO-_2_, using this novel pulse oximeter method, and SaO_2_ will enable continuous, real-time monitoring of these physiological conditions. Thus, improvement and application of this novel concept could aid in diagnosis of medical conditions related to microcirculation dysfunction, such as sepsis and cardiac failure, which are both common in the ICU, as well as length of stay, cost, and mortality.

## Abbreviations

AC: high frequency component of the PPG signal
ADC: analog-to-digital converter
APG: artificial pulse generation
BP: blood pressure
DC: low frequency component of the PPG signal
FFT: Fast Fourier Transform
FIR: finite impulse response
HDEC: Health and Disability Ethics Committee
HR: heart rate
ICU: intensive care unit
IIR: infinite impulse response
IR: infrared light/wavelength
IV: intravenous
LED: light emitting diode
NIRS: near infrared spectroscopy
O_2_E: oxygen extraction
PPG: photoplethysmograph
PO: pulse oximeter
R: modulation ratio
R_Art_: R value derived from AC and DC signals
R_Ven_: R value derived from APG and DC signals
RBC: red blood cell
RD: red light/wavelength
SaO_2_: arterial blood oxygen saturation
SpO_2_: pulse oximeter estimated SaO_2_
SpaO_2_: custom pulse oximeter estimated SaO_2_
SvO_2_: venous blood oxygen saturation
SpvO_2_: custom pulse oximeter estimated SvO_2_
VOT: Vascular Occlusion Test
